# Notes on New Drugs and Preparations for the Sick

**Published:** 1901-12

**Authors:** 


### Notes on New Drugs and Preparations for the Sick.

---

Some new *preparations of pepsin and bismuth*, in various combinations, have been forwarded to us from H. E. Matthews, Clifton, viz.:—

**Pepsinum Liquidum Fortior** (Matthews).—Each fluid drachm peptonises 25,000 grains of coagulated egg albumen by U.S.P. test.

**Pepsinum Liquidum** (Matthews).—Each fluid drachm peptonises 2,500 grains coagulated egg albumen. Dose, 1 to 2 fl. drachms.

**Liquor Bismuthi** (Matthews).—*Neutral* solution of bismuth-ammonium citrate, containing no additional alkaline citrate. B.P. strength. Dose, 1 to 2 drachms.

**Liquor Bismuthi c Pepsino** (Matthews).—Each fluid drachm contains in a concentrated form Pepsinum Liquidum ʒ j., and Liquor Bismuthi ʒ j. Dose, 1 to 2 fl drachms.

**Liquor Bismuthi c̄ Pepsino et Euonymin** (Matthews).—Each fluid drachm contains in a concentrated form Euonymin Liquidum ♏ ij. (= 1 gr. Euonymin).

**Liquor Bismuthi Salicylatis** (Matthews).—Of the Bismuth strength of the B.P. Liquor Bismuthi, but the Bismuth contained as Salicylate derived from *natural* salicylic acid. The product is neutral, and is incompatible with acids and strong alkalies, and with ordinary salts of quinine. Dose, $\frac{1}{2}$ to 2 fl. drachms.

We have always found these preparations reliable, and the last-mentioned offers a particularly useful preparation for cases of gastric catarrh associated with the milder forms of rheumatism.

---

**Guaiacum Resin in Capsule.**—Messrs. Blake, Sandford & Blake, 49 Dover Street, Piccadilly, send us samples of their capsules, each containing five grains of powdered resin, not compressed, and therefore readily diffusible. The value of guaiacum resin is greatly diminished by the difficulty of its administration: when patients will take the mixture made with Pulv. Amygdalæ Co., this is doubtless the most efficient way in which full doses of twenty to thirty grains can be given; but the capsule form will enable many chronic gouty and rheumatic patients to take a small dose frequently.

**Hydrochloride of Heroin.**—Bayer & Co., Elberfeld.—This soluble salt of a very useful opium alkaloid gives an aqueous solution of neutral reaction, suitable for subcutaneous injection in doses of $\frac{1}{12}$ to $\frac{1}{6}$ of a grain.

**Bromogene Health Candles; Health Night Lights** (Fowler's Patents).—Messrs. CHRISTR. THOMAS & BROTHERS, Bristol.—Each candle contains about 1¼ grains of dibromo-naphthaline, equal to about two-thirds of a grain of bromine, so that one-tenth of a grain of free bromine is given off per hour.

Each night-light contains about 1½ grains of the haloid compound: as the night-light burns it diffuses into the air, gradually and uniformly, about $\frac{8}{10}$ grain of free bromine.

They are said to be useful in cases of colds, coughs, influenza, whooping cough, and all infectious diseases; also in bronchitis, asthma, &c. As a mental *placebo* they may doubtless be very effectual, but we question if the amount of bromine liberated is sufficient for any appreciable therapeutic activity.

**Byno-Glycerophosphates; Byno-Hæmoglobin.**—ALLEN & HANBURYS, London.—These new additions have recently been made to the Bynin series of malt preparations. The glycerophosphates have proved themselves of value in building up weakly frames and in restoring vigour to persons enfeebled by wasting illnesses: the bynin combination must provide a concentrated food of the highest degree of nutritive value.

The hæmoglobin compound may well be expected to give better results than the ordinary inorganic preparations of iron. One ounce contains one drachm of the preparation called hæmoglobin, and of this mixture the usual adult dose is half an ounce. The combination is quite palatable, and keeps well.

**Palatinoids—Compound Glycerophosphates.**—Messrs. OPPENHEIMER, SON & CO., 179 Queen Victoria Street, London.—In order to avoid the liability to decomposition of these unstable salts they have been prepared in palatinoids, which, being air-tight, preserve their contents from any decomposition arising from exposure to air. If glycerophosphates have all the virtues attributed to them, surely this must be one of the most useful palatinoids in the makers' lists.

**Mist. Pepsinæ c̄ Bismutho** (HUXLEY). **Glycerophosphates** (HUXLEY), Syrup and Tablets. **Benzo-Kinone,** $C_6 H_6 O_2$. **Kügloïds.**—38A Tamworth Road, Croydon.—The Anglo-American and Continental Pharmaceutical Company send us these samples.

The Pepsine mixture is of the ordinary type, containing pepsine, bismuth, hydrocyanic acid, nux vomica, and chloric ether, with a small dose of morphia. It is an elegant looking preparation of a combination which has a wide range of utility.

The Glycerophosphates are now quite taking the medical and the popular fancy. The combination is founded upon the idea that the spermatic fluid, of which Brown Séquard expected such great things, contains a highly concentrated form of glycerophosphates, and that the urine of neurasthenics contains a quantity of phosphates, also in the form of glycerophosphates. It has been shown by Dr. Albert Robin that glycerophosphates have a tendency to stop the morbid elimination of phosphorus from the body.

The Benzo-Kinone is a non-caustic soluble derivative of guaiacol, dispensed in the form of a syrup elixir, containing ten centigrammes of the salt to the teaspoonful.

The Kügloïds are capsules of 20 centigrammes, containing $2\frac{1}{2}$ centigrammes of glycerophosphate of quinine, dissolved in benzoate of creasote and eucalyptol. They have given good results in cases of bronchitis, influenza, and other lung conditions, such as febrile tuberculosis. They are taken in doses of ten or twelve daily.

**Brand's Nutrient Powder from Raw Meat** (Dence's Patent); **Brand's Fever Food.**—Brand & Co., London.—Meat powders have not been altogether a success. Many have been introduced, but their popularity has been short lived, and patients who are induced to try them once do not usually ask for more. This powder should be more popular: it consists of powdered muscle fibre only, uncoagulated, but sterile and tasteless. One ounce is equivalent in nutritive value to four ounces of fresh lean meat. It may be given in many ways, and one to two ounces daily would suffice for the complete maintenance of the body weight under average conditions of the invalid's life.

The fever food is a combination of essence of beef, egg, and cream, manufactured especially for hot climates.

**Tabloids.**—Burroughs, Wellcome & Co., London.

Quinine and Camphor—Quiniæ bisulphate gr. i., camphor gr. $\frac{1}{5}$.

Quinine, Belladonna, and Camphor—Quin. sulph. gr. $\frac{1}{4}$, ext. belladonnæ gr. $\frac{1}{8}$, camphor gr. $\frac{1}{4}$

Morphine and Emetine—Morphine sulph. gr. $\frac{1}{40}$, emetine gr. $\frac{1}{80}$.

Ophthalmic Tabloid.—Alum gr. $\frac{1}{250}$.

The quinine and camphor tabloid, and that with belladonna, are likely to be of value in the treatment of the early stages of catarrh, when they should be taken every hour.

The morphia-emetine tabloid should be an improvement on the old morphia and ipecacuanha lozenge whenever a sedative and expectorant are required.

The ophthalmic tabloid dissolves readily when placed in the conjunctival sac of the lower eyelid.

**Diazyme.**—Fairchild Bros. & Foster, New York.—This essence is very rich in diastasic power, and may be relied upon to compensate for any deficiency of salivary and pancreatic digestion. It converts farinaceous substances into achroodextrines and maltoses, as is done in the intestines by the natural amylolytic enzyme. To aid intestinal digestion it should be taken between meals, two or three hours after food, in doses of one or two teaspoonfuls. It is an elegant preparation, which might be of much service in many conditions of defective intestinal digestion and mal-nutrition.

